# From δ-aminolevulinic acid to chlorophylls and every step in between: in memory of Constantin (Tino) A. Rebeiz, 1936–2019

**DOI:** 10.1007/s11120-020-00750-x

**Published:** 2020-05-26

**Authors:** Govindjee Govindjee, Donald P. Briskin, Christoph Benning, Henry Daniell, Vladimir Kolossov, Hugo Scheer, Mark Rebeiz

**Affiliations:** 1 Department of Plant Biology, Department of Biochemistry, and Center of Biophysics & Quantitative Biology, University of Illinois at Urbana-Champaign, Urbana, IL 61801 USA; 2 Stress Physiology and Molecular Biology Laboratory, School of Life Sciences, Jawaharlal Nehru University (JNU), New Delhi, 110067 India; 3 Department of Crop Sciences, University of Illinois at Urbana-Champaign, Urbana, IL 61801 USA; 4 Department of Biochemistry and Molecular Biology- Plant Biology, Plant Research Laboratory, MSU-DOE, East Lansing, MI 48824 USA; 5 Department of Basic and Translational Sciences, School of Dental Medicine, University of Pennsylvania, Philadelphia, PA 19104 USA; 6 Carl R. Woese Institute for Genomic Biology, University of Illinois at Urbana-Champaign, Urbana, IL 61801 USA; 7 Department of Biology- Botany, Ludwig-Maximilians-University, 80638 Munich, Germany; 8 Department of Biological Sciences, University of Pittsburgh, Pittsburgh, PA 15260 USA

**Keywords:** Chlorophyll biosynthesis, Aminolevulinic acid, Skin cancer, Photosynthesis, Rebeiz foundation, Lebanon

## Abstract

Constantin A. (Tino) Rebeiz, a pioneer in the field of chlorophyll biosynthesis, and a longtime member of the University of Illinois community of plant biologists, passed away on July 25, 2019. He came to the USA at a time that was difficult for members of minority groups to be in academia. However, his passion for the complexity of the biochemical origin of chlorophylls drove a career in basic sciences which extended into applied areas of environmentally friendly pesticides and treatment for skin cancer. He was a philanthropist; in retirement, he founded the Rebeiz Foundation for Basic Research which recognized excellence and lifetime achievements of selected top scientists in the general area of photosynthesis research. His life history, scientific breakthroughs, and community service hold important lessons for the field.

*It has been my experience that all phenomena can be conveniently classified as dynamic or static phenomena. Dynamic phenomena encompass the present and immediate future, and consist of our ever changing daily actions. Research is a dynamic phenomenon as research scientists carry their daily research and try to build a scientific legacy. What is a bandwagon at the present time may become ordinary in a few years as other bandwagons come into being. Therefore, by its nature dynamic undertakings such as research have a built in transient characteristic. On the other hand, static phenomena such as significant past research discoveries belong in the realm of history.––Rebeiz* (2014a).

## Beginnings

**Fig. 1:**
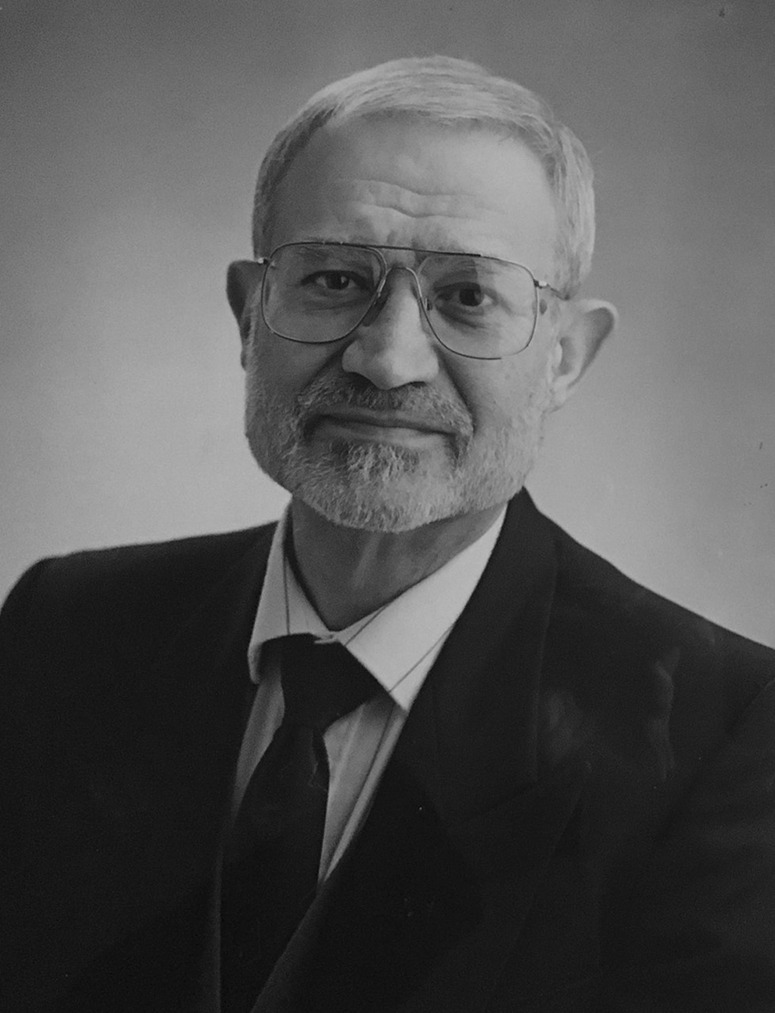
A 1985 portrait of Constantin A. Rebeiz. Source: Archives of the Rebeiz family

Constantin (Tino) A. Rebeiz (Fig. 1) was born in Lebanon in 1936 and lived a life that might not have seemed destined for science. Indeed, his path to a research career was circuitous and serendipitous. Based on information obtained from his family, we know that he grew up in a family of businessmen, surgeons, and poets. However, throughout his youth, he was influenced by a story that was one of his favorites to recount, especially to his son Mark (one of the coauthors).

**Fig. 2:**
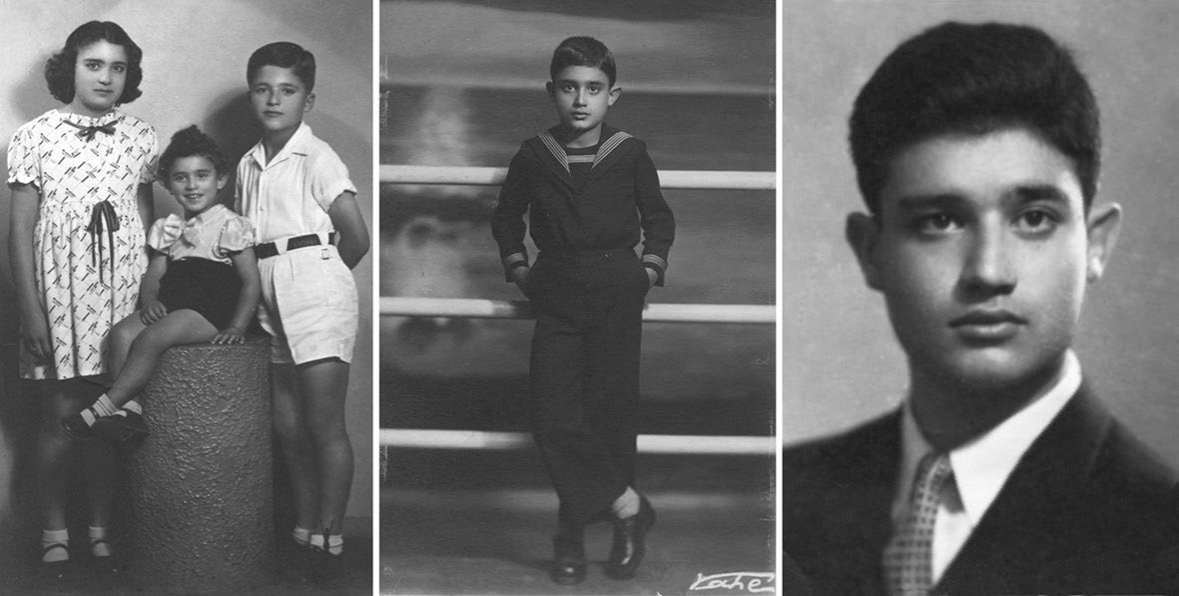
Three photographs of young Tino Rebeiz. Left: when he was 3 years old (sitting in the middle, flanked by his sister May and brother Chucri); middle: when he was 11 years old; and right: when he was 17 years old. Source: Archives of the Rebeiz family

As the story goes, when he was 3 days old, a Moroccan astrologer told his parents to educate him because he would become a well-known scientist, and barring that, he would turn into a criminal. Although the methods were obviously suspect, this episode certainly influenced the scientist he was to become. Figure 2 shows three photographs from his very early life.

Tino attended the American University of Beirut (AUB), where he studied Agricultural Sciences with the goal of aiding the management of a family-owned fruit farm. Upon graduating, in 1959, with distinction, he made the life-changing move to further his education with a Master’s degree (in 1960) in Pomology at the University of California, Davis (UC Davis). Working with Julian Crane (1918–1999), who became a lifelong mentor, he figured out how to make parthenocarpic (seedless) cherries (Rebeiz and Crane 1961). Tino discovered the secret: only treatment with a blend of several plant harmones simultaneously worked to produce seedless cherries. To those of us who knew him well (M.R.), Tino would often lament that seedless cherries never caught on, as folks were used to spitting the pits!

**Fig. 3:**
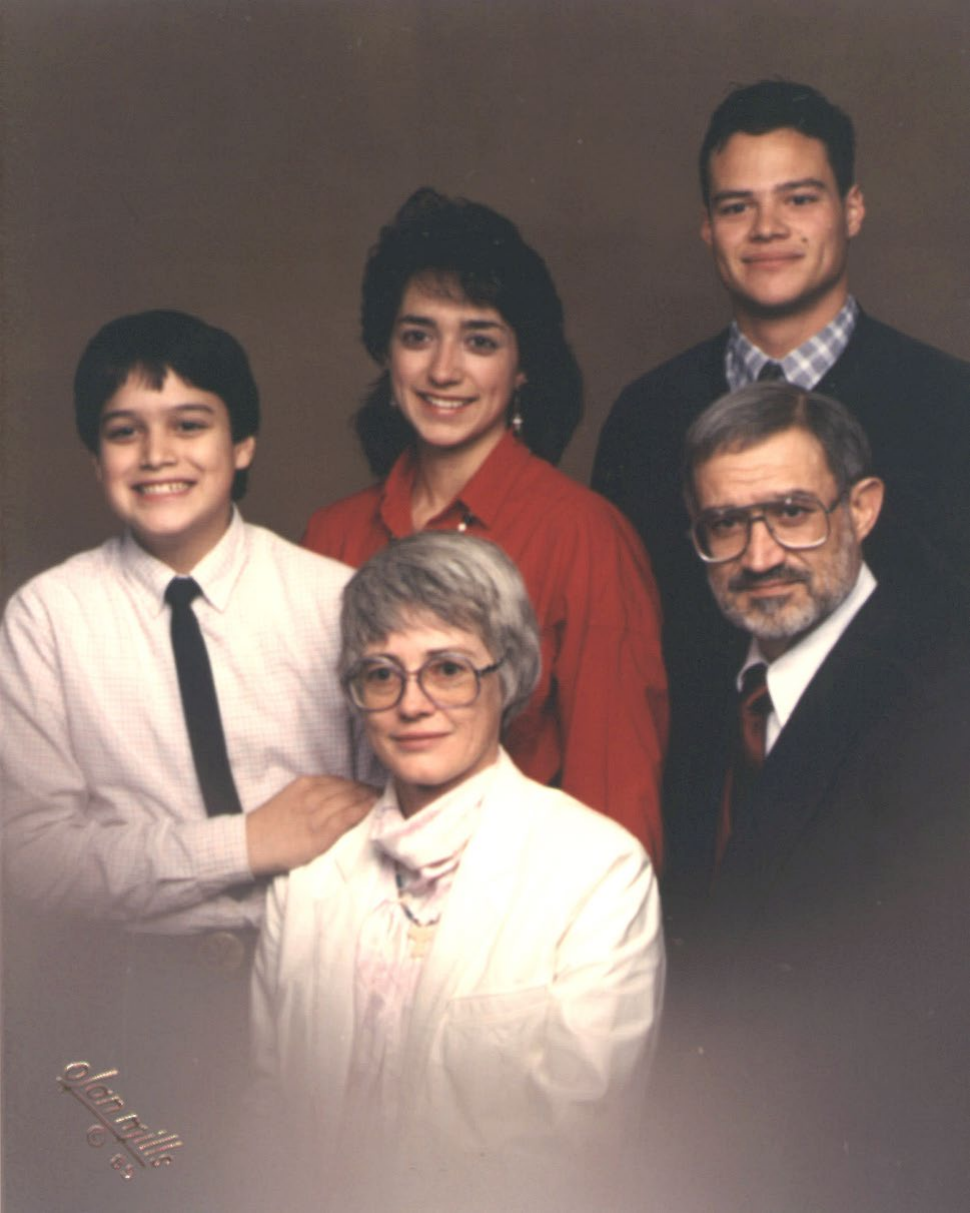
A 1985 photograph of the family of Tino Rebeiz. Back row (left to right): Mark, Natalie and Paul; front row: Carole and Tino. Source: Archives of the Rebeiz family

Tino then started his doctoral research at the UC Davis in the laboratory of Paul Castelfranco (1921–2016), where he characterized the extramitochondrial ß-oxidation of fatty acids in peanut cotyledons (Rebeiz and Castelfranco 1964). During his studies, he met and fell in love with Carole Conness, who he married in 1962, and with her, shared a constant companionship and scientific sounding board until his passing. Upon the completion of his PhD in 1965 from UC Davis, Carole and Tino made the unexpected move to return to Lebanon. Figure 3 shows his photograph, taken in 1985, with Carole and their three children.

The experience of his graduate work at UC Davis galvanized his interest and fulfilled prophecy to continue in the sciences. As a newly minted PhD, Tino took the position of department head at the Lebanese National Research Institute of Tal-El-Amara. There, with great ambition, he developed a department of scientists who were involved in applied soil and leaf analyses. Further, this opportunity gave him the chance to develop a growing interest in basic research, which became a driving motivation of his later career. Looking at the fields of beautiful green plants in the Bekaa (Beqaa) valley, in Eastern Lebanon, he was inspired by all the chlorophyll that must be synthesized to generate the lush landscape. He identified the problem of the biochemical origin of chlorophyll to be one of the most important for plant biology at that time, a problem which he thought could take a lifetime to resolve (Rebeiz 2014a).

In Tal-El-Amara, Tino took the first step of what became a lifelong journey into *chlorophyll biosynthesis* by establishing a cell-free system in which the formation of chlorophyll, as obtained from cotyledon extracts, could be studied (Rebeiz et al. 1970a, b). These experiments built upon his fascination with the rapid greening of etiolated cotyledons (grown in the dark) once exposed to light (Rebeiz 2014a). These studies were primarily performed in his laboratory in Lebanon, but they were finished at UC Davis in the laboratory of Paul Castelfranco as Tino’s family came to the USA after fleeing a brewing civil war in Lebanon. It was at Davis that Tino demonstrated the complete incorporation of ^14^C δ-aminolevulinic acid (ALA) into chlorophyll (Rebeiz and Castelfranco 1971; Rebeiz et al. 1971). He was recognized for this research all over the world, and it paved the way for him to become a faculty member at the University of Illinois at Urbana- Champaign (UIUC), in the USA. We present below some of his key discoveries.

## Multiple biosynthetic routes to chlorophylls

**Fig. 4:**
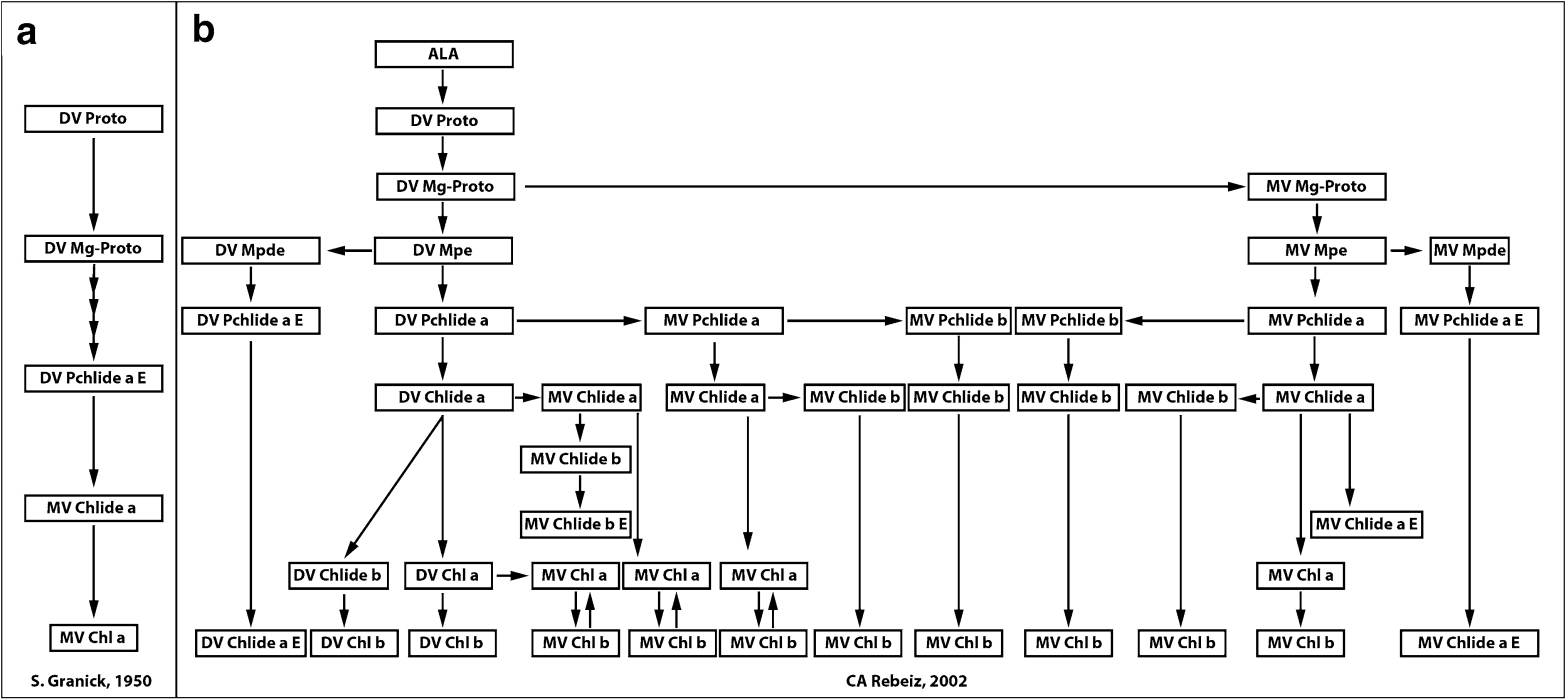
The heterogeneous origins of chlorophyll. **a**: The classic linear, single-branched, Chl *a*/*b* biosynthetic pathway (Granick 1950). **b** Integrated Chl *a*/*b* biosynthetic pathway, adapted from Rebeiz (2002). Arrows joining the divinyl (DV) and monovinyl (MV) branches refer to reactions catalyzed by [4-vinyl] reductases. It is unlikely that all the proposed reactions may be found in a single plant species at all stages of greening. Indeed, based on biosynthetic heterogeneity in the greening process, this figure integrates biosynthetic routes for dark divinyl plants such as cucumber with dark monovinyl plants such as barley. *ALA* 5-aminolevulinic acid, *Chl* chlorophyll, *Chlide* chlorophyllide, *DV* divinyl (vinyl groups at position 2 and 4 of the macrocycle), *MV* monovinyl (vinyl group at position 2 and ethyl group at position 4 of the macrocycle), *Mpe* Mg-Proto monomethyl ester, *Pchlide* protochlorophyllide, *Proto* protoporphyrin lX. Unless preceded by MV or DV, tetrapyrrole names are used generically to designate metabolic pools that may consist of MV and DV components

In 1972, Tino reestablished his research program at UIUC, where he stayed until retirement in 2005. Building from his initial success with cell-free synthesis of chlorophylls, he sought to build support for the biosynthetic pathway, which had been proposed by Sam Granick (1909–1977), one of the greatest chlorophyll biochemists of our time. Granick (1950) initially proposed that this pathway was a linear one (see Fig. 4a). However, Tino and his research group noted failures to establish precursor–product relationships along a linear pathway (Rebeiz et al. 1970b; Mattheis and Rebeiz 1977; Belanger and Rebeiz 1979). This was complemented by observations of multiple ways that the same end product could be generated through distinct intermediates. From these observations, Tino and his team developed a multibranched pathway through which multiple routes led from ALA to chlorophyll (Fig. 4b).

One of us (HS) recalls how Tino was criticized at the time because his conclusions were based mainly on chromatographic and spectrofluorometric methods that provide only indirect structural information. However, with further research, Tino’s ideas turned out to be correct. A breakthrough supporting his view of the parallel biosynthesis of pigments carrying the conventional single vinyl group at the C-4 position and an additional one at the C8 position was provided by Maarib B. Bazzaz (1940–2020; a former PhD student of Govindjee), who identified both mono- and divinyl chlorophylls in a *Zea mays* mutant by Nuclear Magnetic Resonance (Bazzaz and Brereton 1982). An abundance of divinyl chlorophyll was later identified in *Prochlorococcus* sp., a marine bacterium (Ralf and Repeta 1992). In a series of about hundred papers, Tino worked out, over ~ 25 years, a heterogeneous network rather than a linear biosynthesis of chlorophylls *a* and *b*, with preferential paths that depend on the organism, growth conditions including, in particular, light (summarized in Rebeiz (2002); also see Canniffe et al. (2014); Fig. 4). While maintaining and improving spectrofluorometric techniques, Tino Rebeiz was highly respected in his chosen field of research. It is a classical case showing how the potential of seemingly old-fashioned structural methods can be expanded against the odds.

## Exploiting the chemistry of chlorophyll biosynthesis in agriculture and medicine: a lifetime goal

**Fig. 5:**
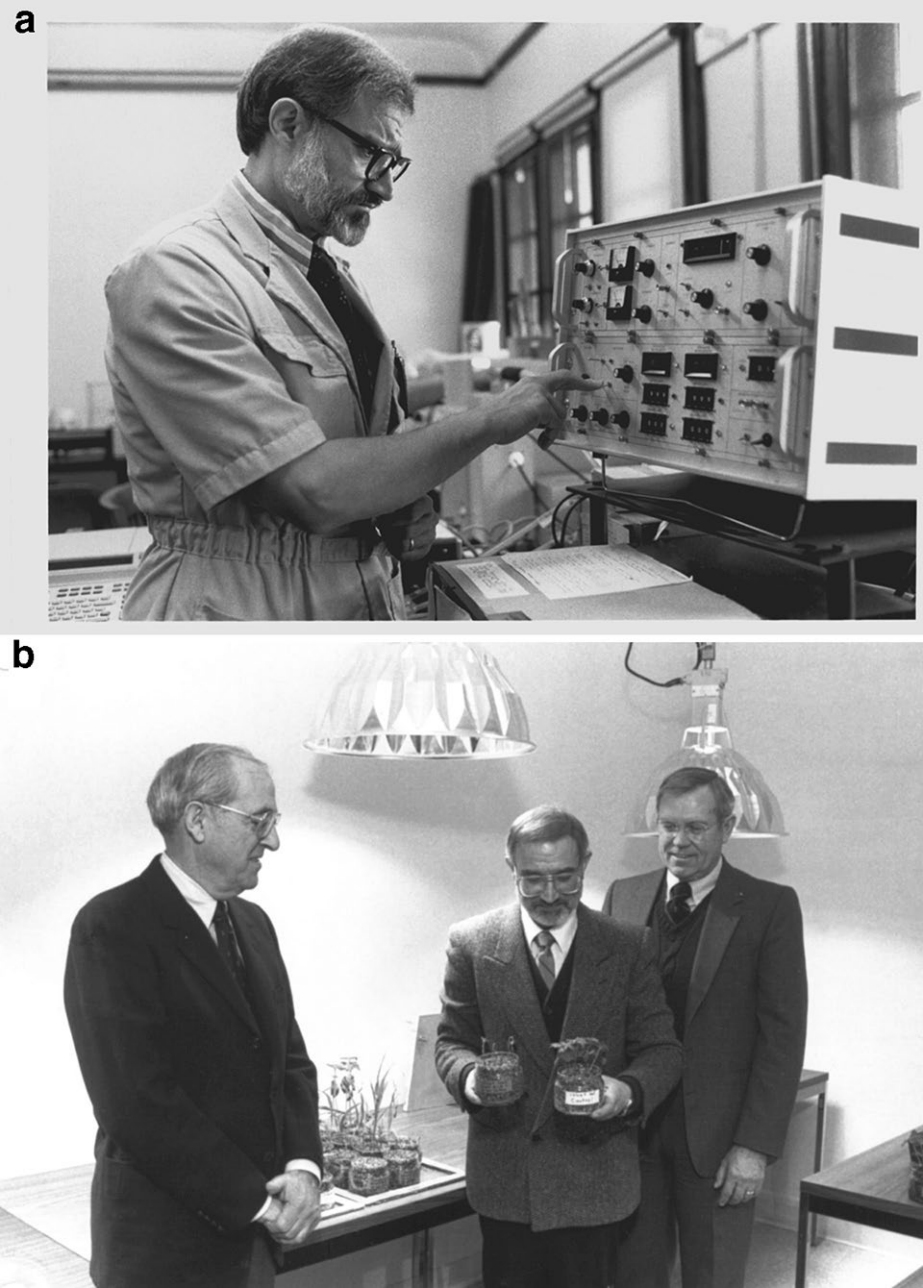
**a** A photograph of Tino Rebeiz doing experiments in his laboratory at the University of Illinois at Urbana-Champaign, 1980s. **b** A 1987 photograph of Tino showing the effects of ALA treatment on plants; on the left is John P. Trebalis (one of the donors for Rebeiz’s research), and on the right is John Campbell, the then Dean of Agriculture of the University of Illinois at Urbana-Champaign. Note that the pot in Tino’s right hand was treated with ALA, while in his left hand was a control. Source: Archives of the Rebeiz family

Tino believed in the importance of translating the results of basic research into applications that would benefit humanity. As his laboratory was developing and evolving a sophisticated understanding of the many ways that chlorophyll is assembled in plant cells, Tino expanded his program into another field of research focused on the photodynamic properties of chlorophylls and their precursors. When not integrated into the photosynthetic apparatus, chlorophylls are highly dangerous molecules that in the presence of light and oxygen generate reactive oxygen species. For example, *Porphyria* is a disorder that results from a buildup of natural chemicals that produce porphyrins in our body; accumulation of porphyrins in the skin sensitizes the patient, in particular to blue light (Phillips 2019). Likewise, treatment of plants with ALA causes accumulation of excess chlorophylls that, upon irradiation, causes cell death (Rebeiz et al. 1984). The main benefit of developing this technology was that ALA is an amino acid that would be easily biodegradable and much more environmentally friendly than commercial alternates. Based on the observation that ALA accumulation is greatly enhanced by bipyridyls (Duggan and Gassman 1974), Tino explored this and other chemicals as modulators in combination with ALA to be economic photodynamic herbicides. He published this exciting research in a series of papers beginning in 1984 (Rebeiz et al. 1984). Further, he exploited the differential responses of different plant species that he had worked out during biosynthetic studies on them, in particular, the agriculturally relevant higher sensitivity of dicots versus monocots (Rebeiz et al. 1984, 1990). However, and unfortunately, the price of ALA is forbidding for using it as a photodynamic herbicide. Eventually, potent inhibitors of protoporphyrinogen oxidase, such as diphenyl-ethers, that circumvent the use of costly ALA (Duke et al. 1991) were found to be much more economical, but that is another story. Figure 5a shows Tino doing experiments in his laboratory in Urbana, Illinois, and in Fig. 5b, he is showing his experimental plants to the visitors to his laboratory.

Inspired by Tino’s work on photodynamic herbicides, several medical researchers contacted him regarding the possibility of using ALA technology to treat cancer (Rebeiz 2014b). This is because chlorophyll and heme share biochemical steps from ALA to protoporyphrin IX. Thus, treatment of cancerous tissues, particularly non-invasive skin cancers, would convert topically applied ALA into porphyrins, which could kill these cells upon UV exposure (Kennedy et al. 1990). Further research by Tino and his daughter Natalie (Rebeiz et al. 1996), on Photodynamic Therapy, using in situ generated sensitizers to the tetrapyrrole precursor, has led ALA to be used in an established clinical method in this area (Agostinis et al. 2011). The above speaks highly of his contributions in adapting basic research leading to applications for our benefit.

## The Rebeiz lab winds down

One of us (D.P.B.) remembers the last 6 years of Rebeiz’s academic career at UIUC, when they had a wonderful opportunity to interact closely with each other. Briskin found Tino to be a talented and highly-spirited scientist in both research and teaching. We note that the two shared a common laboratory space; while many researchers in this position would simply divide up the space and work independently, it was Tino's idea to use this move as an opportunity to develop a collaborative research group that became the "*Laboratory of Plant Biochemistry and Photobiology*" (LPBP) at UIUC. Over the years that LPBP functioned, this association provided an exciting forum for discussions, interaction on fundamental plant research, lab group meetings, and collaboration between these two scientists. Under Tino's influence, this association deeply enriched the research experience of students and staff in Briskin’s group.

## The human connection: Tino as a mentor

Over the course of Tino’s 33-year career at the UIUC, the Rebeiz lab was a lively environment that expanded and contracted with the winds of funding support from the National Science Foundation, and private funds from the French pharmaceutical company Rousell for his applied work on herbicides. During this time, Tino Rebeiz trained a large number of postdoctoral researchers, as well as Master’s and PhD students. Below, we present a few personal reminescences of Tino Rebeiz.

## Henry Daniell recalls

When I was in Prof. Rebeiz’s lab during 1980–1982 as a postdoctoral fellow, I was fortunate to investigate chlorophyll biosynthesis and chloroplast development in vitro. I was thrilled to publish our research on in vitro chlorophyll biosynthesis at rates higher than in nature, and in vitro synthesis of macro-grana for the first time (Daniell and Rebeiz 1982). This research was continued when I became an independent faculty member at Washington State University, where isolated etio-chloroplasts were first used to express “foreign” genes, resulting in a breakthrough publication (Daniell and McFadden 1987), and we obtained the first and the oldest patent in the field of chloroplast genetic engineering (US patent # 5,693,507). Today, the chloroplast biotechnology field has grown to such an extent that there are regular Gordon Research Conferences on this topic, and research in this area has produced commercial enzyme products (Daniell et al. 2019; Kumari et al. 2019) and low-cost drug development (Daniell et al. 2016).

I spent my first Thanksgiving at Tino’s home in 1980 and fondly recall numerous other occasions, including dinners at the Rebeiz foundation award ceremonies in recent years. Beyond academic advances, Tino taught his associates several valuable lessons. I will never forget his advice that only “*the bird that flies high gets shot*,” based on his own experience of peers stabbing his grants or publications. As a foreign-born US citizen, he also shared the challenges he faced in advancing his career, which motivated me not to give up when I faced such adversity. He truly cared about his lab colleagues. Just to give an example, he once observed a small tear in my shirt; he stood up, walked towards me and patched it with scotch tape. It was wonderful for me to advance his dream of “leveraging” chloroplasts to save lives or develop affordable products used in daily life.

## Vladimir Kolossov added

I have had the privilege of knowing Tino for over 20 years from the time I joined his lab as a postdoctoral research associate in January 1996. Yet, he was not a stranger to me even before that time. Tino was a world-recognized scientist when I, as a graduate student, was attending research seminars at the Institute of Photobiology, at the Academy of Sciences of Belarus, led by Alexander A. Shlyk (1928–1984). In the 1980s, I was introduced to the scientific merits of Rebeiz’s research, who was considered a major authority in the field of tetrapyrrole biochemistry. Tino knew many scientists from my institute, including Natalia Averina, who closely followed his research and whose lab I had joined after obtaining my PhD in Molecular Biology. Shortly thereafter, I was invited by Tino to continue my training in chloroplast biogenesis, specifically vinyl reductases at his UIUC lab. At the time I joined Tino’s lab, he was synthesizing accumulated knowledge to propose a unified multibranched chlorophyll biosynthetic pathway (Fig. 4). My work supported this concept and provided important details (e.g., Kolossov and Rebeiz 2010).

Tino was a great mentor and his high ethical standards were exemplified during our publication of research on a controversial topic—occurrence of protochlorophyllide *b* in etiolated plants (grown under dark conditions). I had read a paper (Reinbothe et al. 2003), which claimed the occurrence of a specific metabolite from the chlorophyll *b* biosynthetic pathway that was well established to be absent in etiolated plants. I immediately decided to repeat the published extraction procedure, but without any success. Straight away, I told Tino about the controversial results and my desire to publish our data, as I was fully confident in their validity. Tino supported my enthusiasm but disclosed that the corresponding author of the conflicting data had contacted him a year earlier asking him for advice before submitting their results for publication. Tino, despite his skepticism, had suggested the other researcher to go ahead and publish the results if he was fully confident in the results. Tino was always frustrated by authors who published only the experimental data which supported their previous results and preconceived notions. Obviously, Tino was uneasy in publishing our contradicting results, but once again, he never hesitated to initiate the scientific debate and publish data he trusted completely. Our manuscript (Kolossov and Rebeiz 2003) was submitted and one of the reviewers acknowledged that his lab as well had tried to reproduce the published data of the other research group, but without success. Time of course is the best judge, but Tino was always open for discussion on any conflicting topic.

As I reflect on the time I worked with Tino in his lab as a postdoctoral research associate, I can see today the incredible amount of experience I had gained from his guidance and the invaluable knowledge I attained from my time in his lab. Those of us who worked with him and benefited from his companionship and insight also knew him to be an extraordinary person with an amazing grasp of science, a gift for designing and executing astonishing experiments, and an exceptionally strong moral concern that science be done to benefit everyone.

## Baishnab C. Tripathy (of India) added

Beginning December 1983, I worked with Professor Constantin A. Rebeiz on the heterogeneity of the chlorophyll biosynthesis pathway. Although chlorophyll in flowering plants is monovinyl (MV), it also has a divinyl (DV) precursor isoform. Thus, I quantified these intermediates from their mixtures, using both room and low temperature (77 K) fluorescence spectra, and showed the presence of intermediates such as protoporphyrin IX, Mg-protoporphyrin IX, and protochlorophyllide. We fed isolated etio-chloroplasts with different MV and DV chlorophyll biosynthesis intermediates, and demonstrated that, indeed, there are two parallel MV and DV pathways for chlorophyll biosynthesis. These results were published in high-impact journals (see, e.g., Tripathy and Rebeiz 1985, 1986, 1988). Today, due to the painstaking efforts of Tino Rebeiz and his outstanding coworkers, it is widely accepted that the chlorophyll biosynthesis pathway is indeed branched and vinyl reductase is responsible for this biosynthetic route (see Fig. 4). In 1987, I joined Jawaharlal Nehru University (JNU), New Delhi, as a faculty member in its School of Life Sciences (SLS). However, my interaction with Tino continued and I visited his lab several times. He had great parties on July 4 (the US Independence Day); on Christmas, and on the New Year.

Although Tino was often misunderstood by some, he was very good at heart and had an open mind towards science and society. He always strived to achieve academic excellence. He believed in promoting brilliant people and recognizing their contributions to science (see, e.g., Tables 1 and 2). I had also a very good interaction with his children: Paul, Natalie and Mark (one of the authors of this Tribute). I vividly remember Carole Rebeiz for her affection and unhindered access to her home. Further, I was very happy that Tino allowed me to do some experiments for NASA (National Aeronautics and Space Administration) while I was in his laboratory.

**Table 1:** Names of RFFBR lifetime achievement awardees

1.	Govindjee, 2006, University of Illinois at Urbana-Champaign (UIUC), IL, USA ( see: https://www.life.illinois.edu/govindjee/awardsandhonors.html; Rebeiz et al. 2007))
2.	^#^Paul Castelfranco (1921–2016), 2007, formerly at the University of California at Davis, CA,USA (obituary unavailable)
3.	^#^Andrew A, Benson (1917–2015), 2008, formerly at the University of California at Berkeley, CA, USA (see, e.g., Buchanan et al. 2016)
4.	^#^Diter von Wettstein (1929–2017), 2009, formerly at the Washington State University, Seattle, WA,USA (see e.g., Hoober 2017)
5.	William Ogren, 2010, United States Department of Agriculture (USDA), and UIUC, Urbana, IL, USA (see Portis and Govindjee 2012)
6.	Bob Buchanan, 2011, University of California at Berkeley, CA, USA (see http://www.life.illinois.edu/govindjee/honorsfrom.html for a presentation on Buchanan)
7.	^#^Andre Jagendorf (1926–2017), 2012, formerly at Cornell University, Ithaca, NY, USA ( see e.g., Govindjee 2019)
8.	Wolfgang Junge, 2012, University of Osnabrueck, Germany (see http://www.life.illinois.edu/govindjee/honorsfrom.html for a presentation on Junge)
9.	^#^Roland Douce (1939–2018), 2013, formerly at the University of Grenoble, France (see e.g., Joyard and Lichtenthaler 2019)
10.	Robert Blankenship, 2013, Washington University, St. Louis, MO, USA(See http://www.life.illinois.edu/govindjee/honorsfrom.html for a presentation on Blankenship)
11.	Hartmut Lichtenthaler, 2014, Karlsruhe Institute of Technology, Germany (See http://www.life.illinois.edu/govindjee/honorsfrom.html for a presentation on Lichtenthaler)
12.	Pierre Joliot, 2014, Centre National Recherche Scientifique, Paris, France (See http://www.life.illinois.edu/govindjee/honorsfrom.html for a presentation on Joliot)

^#^Deceased

**Table 2:** List of RFFBR paper awards

1.	Forster B, Mathesius U, Pogson BJ (2006) Comparative proteomics of high light stress in the model alga Chlamydomonas reinhardtii. Proteomics 6: 4309–4320
2.	Chew AG, Bryant DA (2007) Characterization of a plant-like protochlorophyllide a divinyl reductase in green sulfur bacteria. J Biol Chem 282: 2967–2975
3.	Moulin M, McCormac AC, Terry MJ, Smith AG (2008) Tetrapyrrole profiling in Arabidopsis seedlings reveals that retrograde plastid nuclear signaling is not due to Mg-protoporphyrin IX accumulation. Proc Natl Acad Sci U S A 105: 15178–15183
4.	Bräutigam K, Dietzel L, Kleine T, Stroher E, Wormuth D, Dietz KJ, Radke D, Wirtz M, Hell R, Dormann P, Nunes-Nesi A, Schauer N, Fernie AR, Oliver SN, Geigenberger P, Leister D, Pfannschmidt T (2009) Dynamic plastid redox signals integrate gene expression and metabolism to induce distinct metabolic states in photosynthetic acclimation in Arabidopsis. Plant Cell 21: 2715–2732
5.	Liu C, Young AL, Starling-Windhof A, Bracher A, Saschenbrecker S, Rao BV, Rao KV, Berninghausen O, Mielke T, Hartl FU, Beckmann R, Hayer-Hartl M (2010) Coupled chaperone action in folding and assembly of hexadecameric Rubisco. Nature 463: 197–202
6.	Kanady JS, Tsui EY, Day MW, Agapie T (2011) A synthetic model of the Mn-Ca subsite of the oxygen-evolving complex in photosystem II. Science 333: 733–736
7.	Kikuchi S, Bedard J, Hirano M, Hirabayashi Y, Oishi M, Imai M, Takase M, Ide T, Nakai M (2013) Uncovering the protein translocon at the chloroplast inner envelope membrane Science 339: 571–574
8.	Liu H, Zhang H, Niedzwiedzki DM, Prado M, He G, Gross ML, Blankenship RE (2013) Phycobilisomes supply excitations to both photosystems in a megacomplex in cyanobacteria. Science 342: 1104–1107

I was delighted that Tino had appointed me as a permanent member of the Executive Board of the Rebeiz Foundation that he established after I had left the University of Illinois at Urbana-Champaign. Contributions of Tino Rebeiz to chlorophyll biosynthesis and photodynamic herbicides is immense and he will be remembered forever by the scientists working in the field. We all miss him dearly.

## Raj Prasad (of Canada) added

I am extremely sorry to hear about the passing away of Constantin (Tino) Ribiez. I knew him during 1961–1962, while we both were at the University of California (UC) -Davis. Tino was working with Paul Castefranco, whereas I was in the Lab of Alden Crafts (working on ‘Herbicides’). We interacted with each other in the labs, in the greenhouses, and at seminars. I would like to mention that Tino’s research indeed inspired me in some of my own research. He was not only a hard-working scientist, but a highly enthusiastic and a creative one. What we had in common was that both of us were ‘foreign-born’; I liked his attitude to go back to his country (Lebanon) and serve there, which he did at that time.

## Constantin A. and Carole C. Rebeiz foundation for basic research

Following retirement in 2005, Tino's intense passion for plant research continued with his establishment of the “The C. A. and C. C. Rebeiz Foundation for Basic Research” (RFBR), which served to encourage and promote advanced research in photosynthesis and enrich the scientific community. Two of the coauthors of this Tribute (C.B. and G.G.) summarize our memories of collaborating with him on this endeavor.

Leading up to the establishment of the RFBR, the “First International Symposium on Chloroplast Bioengineering” was held at the UIUC, during May 2–May 7, 2005. Participants at this conference contributed chapters to a very thorough book”The Chloroplast: Basics and Applications” (Rebeiz et al. 2010). Several of the participants of this meeting and some of the editors of the book were invited by Tino to become members of the governing board of the RFBR. In 2014, the last year the RFBR met in Urbana-Champaign, the governing board included: Constantin A. Rebeiz (President, RFBR), 3 of the authors of this Tribute (Christoph Benning, Henry Daniell and Govindjee), Donald Bryant (Pennsylvania State University), William Lucas (University of California Davis), Harald Paulsen, (University of Mainz, Germany), Archie Portis (University of Illinois at Urbana-Champaign), Tom Sharkey (Michigan State University), Wolfgang Junge (University of Osnabrueck, Germany), Baishnab C. Tripathy (Jawaharlal Nehru University, India) and Carole Rebeiz (Secretary, RFBR).

**Fig. 6:**
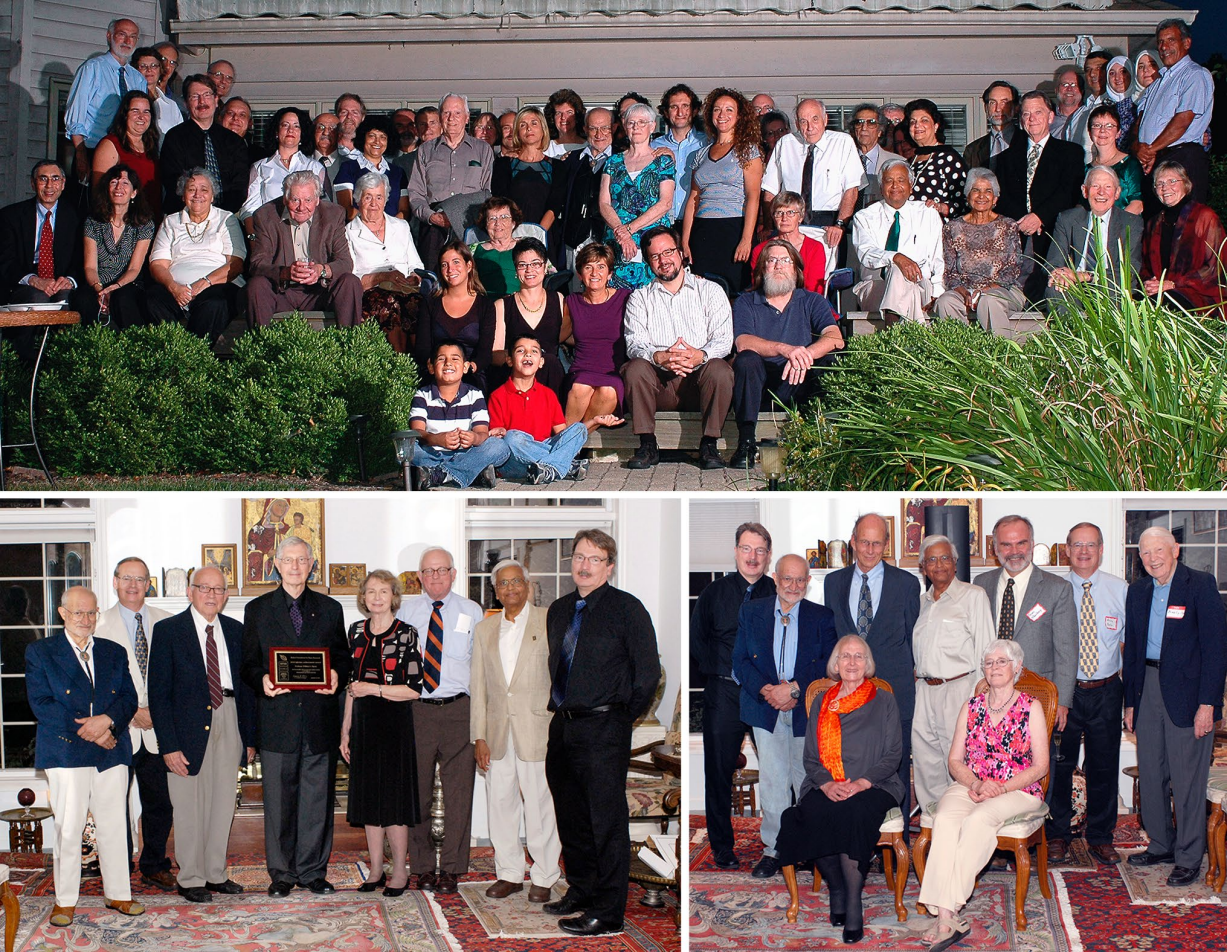
Three group photographs from the Rebeiz Foundation for Basic Research (RFBR) Award ceremonies (2010–2012). Top: A large group of guests gathered to honor, in 2010, Diter von Wettstein (1929–2017); he is standing in the back row (on the right side of the photo), wearing a half-sleeve white shirt & a black tie. Carole & Tino Rebeiz are third and fourth on his right. On the left of von Wettstein is Tino’s friend Raheel (a well-known artist whose paintings adorn Rebeiz’s home) and his wife Mastura (Professor of Home Economics at UIUC). Below this couple is Govindjee (wearing a green tie) and his wife Rajni. Carole (wearing a green dress) and Tino Rebeiz are third and fourth on Von Wettstein’s right. Among the other guests, we mention just a few: Christoph Benning is on the top left side of the photograph; he is wearing a black shirt and a grey tie; fifth on his left is Dennis Buetow (wearing a open-collar brown shirt, with a black glass case in his pocket). Bottom (left): A photograph of some of the guests honoring William (Bill) Ogren (in 2011); left to right: Tino Rebeiz; Archie Portis; the late David Krogmann (1934–2016); Bill Ogren; Carolyn Ogren (Bill’s wife); Jack Widholm; G.Govindjee; and Christoph Benning. Bottom (right): A photograph of some of the guests honoring Bob Buchanan (in 2012); Standing: left to right: Christoph Benning; Tino Rebeiz; Bob Buchanan; G. Govindjee; Tom Sharkey; Archie Portis; ----; Sitting (left to right): Melinda Buchanan; Carole Rebeiz. Photo credit: Laurent Gasquet

**Fig. 7:**
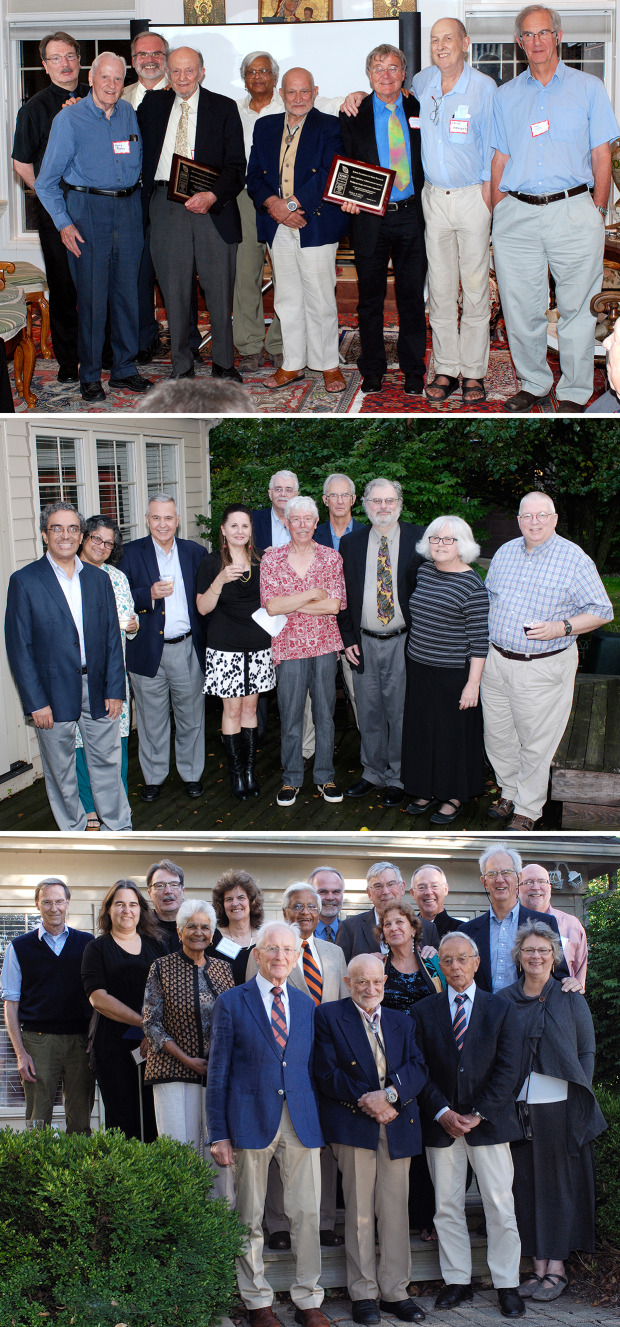
Group photographs from Rebeiz Foundation for Basic Research (RFBR) Award ceremonies (2013–2015). Top: Honoring Andre Jagendorf and Wolfgang Junge (in 2013); left to right: Christoph Benning; Dennis Buetow; Thomas (Tom) Sharkey; Andre Jagendorf; Govindjee Govindjee; Tino Rebeiz; Wolfgang Junge; the late Colin Wraight (1945–2014; see Govindjee et al. 2016); and Tony Crofts. Middle: Honoring Roland Douce and Robert (Bob) Blankenship (in 2014). Front row (left to right): Himadri Pakrasi; Maitrayee Bhattacharyya; ----; ----; Roland; Douce; Bob Blankenship; Liz Blankenship; Don Bryant. Back row: Tony Crofts (just behind Blankenship). Bottom: Honoring Hartmut Lichtenthaler and Pierre Joliot (in 2015). First row (L to R): Hartmut Lichtenthaler; Tino Rebeiz; Pierre Joliot; Christine Yerkes. Second row (L to R): Rajni Govindjee; Govindjee Govindjee (wearing an orange and blue tie); Hanni Cramer-Third row ( L to R): Bruce Diner; Susanne Hoffmann-Benning; Christoph Benning; -----; Tom Sharkey; William (Bill) Cramer; -------; Antony (Tony) R. Crofts (behind Joliot and Yerkes); Photo credit: Laurent Gasquet

The RFBR governing board was responsible for nominating and voting on the awardees for the LifeTime Achievement Award given for outstanding contributions to photosynthesis, chloroplast research, and plant biochemistry. Ten RFBR LifeTime Achievement Awards were presented (see Table 1 for the list) at annual dinner ceremonies at the RFBR headquarters, at Tino’s home. These ceremonies were dignified, with testimonials presented by colleagues to the gathered guests, including members of the board and friends of the RBFR. Many of us will miss these annual meetings and the hospitality and generosity of Carole and Tino Rebeiz, who prepared wonderful Lebanese cuisine and entertained their guests in their beautiful home, furnished with art and artifacts. Figures 6 and 7 show photographs of some of the award ceremonies, randomly selected by us. The first one to be honored was Govindjee (Rebeiz et al. 2007) and the last to be honored were Pierre Joliot and Hartmut Lichtenthaler. William (Bill) Ogren, a long-time colleague at the UIUC in photosynthesis related research and the 2010 awardee, states on behalf of all the awardees: “*We remain forever grateful to Tino and the Board of Directors of his Foundation for our recognition*”. Further, the late Andre Jagendorf, a 2012 awardee, had beautifully expressed his gratitude to the Rebeiz family in an e-mail, as follows.

Dear Carole and Tino: As our plane flew away from the Willard airport, I looked down and I saw 8 or 9 small lakes. One of them had a long peninsula, and at the end of the peninsula stood a fairly large wonderful looking house. I thought it must be yours, for sure; and it was a house in which Jean and I spent the most fabulous week-end in our life together! Your hospitality was overwhelming, and the entire experience was totally unique and wonderful for us. We really had not dreamed of anything like the warmth, the art treasures, the depth of the way in which we were made comfortable and welcomed. We can say "Thank You" over and over, but our words can only express the surface of the pleasures you gave us.—André, October 1, 2013

The RFBR also gave an annual prize for The Best Research Paper in the field of *Chloroplast Biochemistry and Molecular Biology.* The recipients of these awards (see Table 2) were also nominated and voted on by members of the RFBR governing board.

## Appreciating a heterogeneity of ideas in science, and the scientists who perform it

Tino’s scientific story has many lessons that are likely to remain relevant in the foreseeable future to the scientific research community. He represented much of the diversity and heterogeneity that the scientific community is struggling to enrich in its ranks today. He came into academia through a non-traditional path as a non-US-born youth whose family did not fully appreciate the merits of curiosity-driven research. His career path ventured away from a traditional academic trajectory as he developed a research program in a Lebanese government lab. He struggled to maintain a position in academic research as he fled conflicts in his home country and worked part-time as an instructor at Fresno State University (now Cal State University, Fresno). The struggles he overcame are all too familiar to many who are concerned about their job prospects.

One of the greatest benefits derived from enhancing diversity is the heterogeneity of perspectives that groups from different backgrounds bring to a problem. Tino exemplified this in everything he did. His ideas challenged long-held views of chlorophyll biosynthesis, and extended to in-depth discussions that questioned how we perform and report science as a community (as exemplified by the opening quote in this Tribute). To one of us (H.S.), he was a solitary scientist, and stubborn but highly creative sparring partner. He lamented at the endlessly elaborated administrative structures in most Universities, and their focus on erecting new buildings and collecting overhead costs from federal research grants at the expense of fostering the creativity and curiosity of independent thinkers in their faculty. And he was concerned that the unusual fast rise of molecular biology may dampen progress in the study of highly complex biochemical and biophysical phenomena.

Tino recognized through his life work, and especially in retirement how scientific communities need to encourage and foster progress by acknowledging excellence of the past, the present, and even future researchers. To one of us (M.R.), he was an inspiration to develop one’s own scientific legacy, weathering the challenges of a difficult career path. With his passing, Tino's deep passion, drive and spirit for conducting, encouraging, and supporting fundamental plant science research will be greatly missed.

**Fig. 8:**
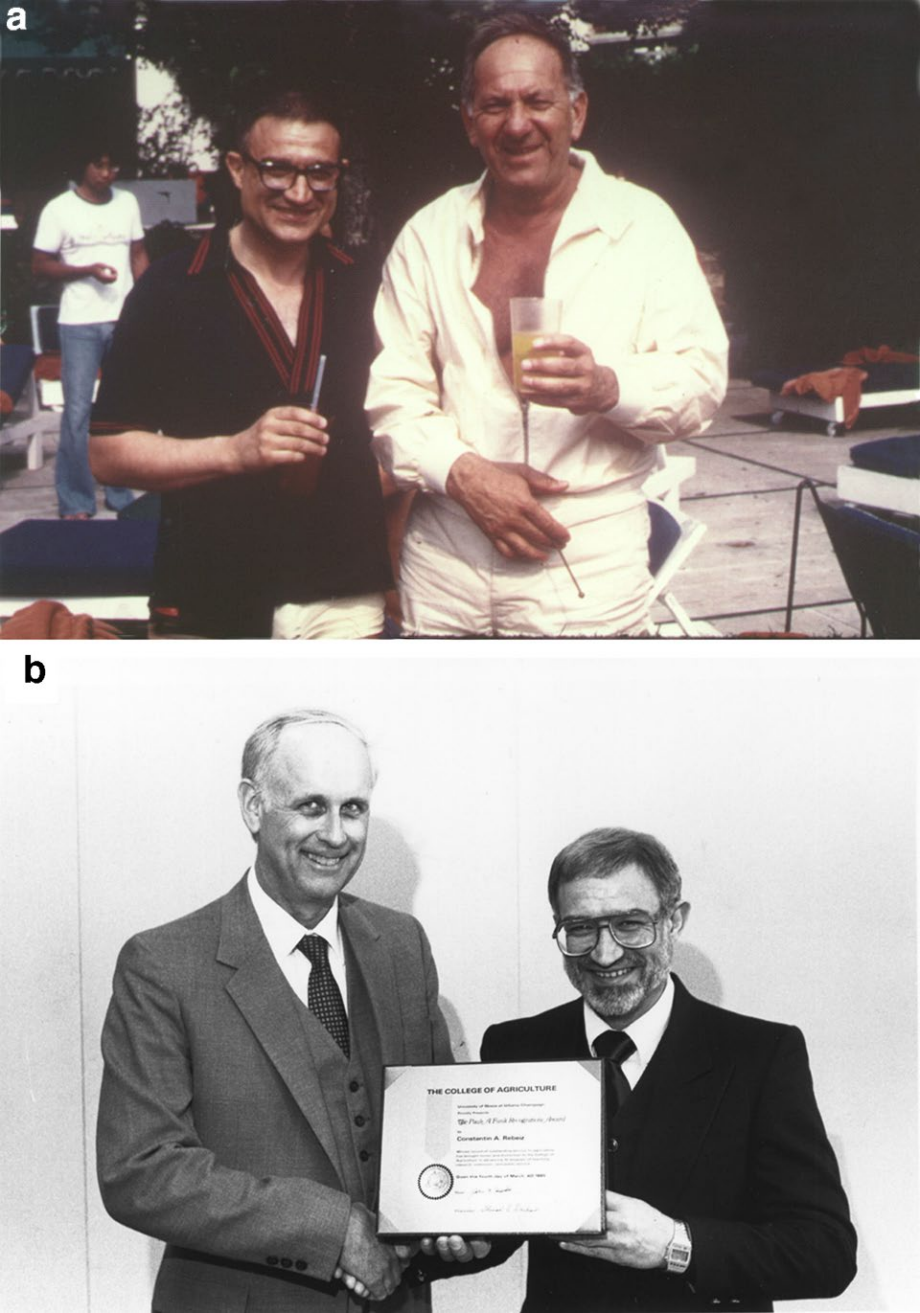
**a** Tino with a famous actor Jack Klugman in Halkidiki, Greece, ~ 1975. **b** Tino receiving, in 1985, the Paul A. Funk Award at the University of Ilinois at Urbana-Champaign (https://awards.aces.illinois.edu/award-funk.cfm). Source Archives of the Rebeiz family

We end this Tribute with two photographs of Tino, one with a famous actor Jack Klugman; and another receiving the Paul A. Funk Award at the University of Ilinois at Urbana-Champaign (see Fig. 8).
